# The Use of Fibrin Clot During Meniscus Repair in Young Patients Reduces Clinical Symptom Rates at 12-Month Follow-Up: A Pilot Randomized Controlled Trial

**DOI:** 10.3390/medicina61091616

**Published:** 2025-09-07

**Authors:** Viktorija Brogaitė Martinkėnienė, Donatas Austys, Andrius Brazaitis, Aleksas Makulavičius, Tomas Aukštikalnis, Ilona Dockienė, Gilvydas Verkauskas

**Affiliations:** 1 Faculty of Medicine, Vilnius University, LT-03101 Vilnius, Lithuania; 2 Department of Children’s Orthopedics and Traumatology, Vilnius University Hospital Santaros Klinikos, LT-08406 Vilnius, Lithuania; 3 Department of Public Health, Institute of Health Sciences, Faculty of Medicine, Vilnius University, LT-03101 Vilnius, Lithuania; 4 Department of Radiology, Nuclear Medicine and Medical Physics, Faculty of Medicine, Vilnius University, LT-03101 Vilnius, Lithuania; 5 Clinic of Rheumatology, Orthopaedics Traumatology and Reconstructive Surgery, Faculty of Medicine, Vilnius University, LT-03101 Vilnius, Lithuania; 6 Department of Rehabilitation, Physical and Sports Medicine, Institute of Health Sciences, Faculty of Medicine, Vilnius University, LT-03101 Vilnius, Lithuania; 7 Institute of Clinical Medicine, Department of Children’s Emergency Medicine, Intensive Care and Anesthesiology, Faculty of Medicine, Vilnius University, LT-03101 Vilnius, Lithuania; 8 Clinic of Gastroenterology, Nefrourology and Surgery, Faculty of Medicine, Vilnius University, LT-03101 Vilnius, Lithuania

**Keywords:** knee, meniscal repair, fibrin clot, young patients, randomized trial

## Abstract

*Background and Objectives:* The menisci are crucial fibrocartilaginous structures of the knee joint and have to be repaired in case of a tear. However, not all meniscal tears heal, even in young patients. Fibrin clot (FC) started to be used to reduce the failure rates following meniscus repair. The purpose of this study is to evaluate and compare outcomes after isolated arthroscopic meniscal repair augmented with FC versus without FC. *Materials and Methods:* Fifty-nine patients aged under 19 with isolated meniscal tears were randomized into two groups: one group underwent the meniscal repair with FC (FC-augmented), and the other group did not receive FC (control). The evaluation and comparison between the groups based on FC augmentation included secondary arthroscopy rates, patient-reported outcome measures (Pedi-IKDC, Lysholm, and Tegner), and clinical and radiological (MRI) assessments at a median follow-up of 12 months. *Results:* No statistically significant difference was observed between FC-augmented and control groups in Pedi-IKDC, Lysholm, and TAG scores, or following clinical and radiological (MRI) evaluation. Patients in the FC-augmented group reported fewer clinical symptoms at the final follow-up across unstable and demanding (bucket-handle and complex) tear type subgroups (*p* = 0.012 and 0.041, respectively). Overall, nine revision arthroscopies occurred in both groups (2 and 7, respectively), all across bucket-handle and complex tears with no significant difference between the FC-augmented and control groups (*p* = 0.072). *Conclusions:* This pilot study found that FC usage during meniscal repair reduces clinical symptoms for patients with unstable, bucket-handle, or complex meniscal tears at the final follow-up of 12 months postoperatively. Nonetheless, no statistically significant differences were observed within the other outcome measures between the FC-augmented and control groups and subgroups based on meniscal tear types. Level of evidence: Level II.

## 1. Introduction

The menisci are very important fibrocartilaginous structures of the knee joint, which serve numerous crucial functions, such as load transfer, cartilage protection, shock absorption, lubrication, and stabilization of the knee joint [1,2,3]. Meniscal tear is a widely prevalent musculoskeletal disorder in adults, and its incidence has dramatically risen, even in the adolescent population, over the last decades [4,5,6,7]. It has been proven that meniscal tissue loss due to injury or resection causes early-onset knee osteoarthritis [8,9,10]. As a result, meniscal repair, rather than the outdated meniscectomy, became essential as the first-line treatment, especially in younger active patients [5,11,12]. According to studies, the clinical outcomes after isolated meniscus repair show an overall failure rate of approximately 20–40% in adults [13,14,15] and from 0% to 70% in children [16,17,18], indicating that not all meniscal tears heal, even in children. This could be linked to the anatomic characteristics of the meniscus vascularity and its capacity for healing [2,19,20]. At birth, the menisci are vascularized throughout their substance, but this vascularity gradually reduces centrally until it reaches the adult pattern by about 10 years of age [6,21]. According to the studies, the meniscus is vascularized mostly in the outer-peripheral (red–red zone) area and just partially in the center area (red–white zone), with the inner part being avascular (white–white zone) from about 10 years of age, and this vascular distribution significantly impacts the healing capacity, resulting in decreased healing potential in the avascular zone of the meniscus. [19,22]. Furthermore, meniscal tear complexity, chronicity, and instability may all impact the healing following a meniscal repair procedure [23].

Therefore, in recent decades, many biological strategies have been presented to augment meniscal repair, including Platelet-Rich Plasma (PRP), fibrin clots, mesenchymal stem cells (MSCs), bone marrow stimulation, meniscal scaffolds, and meniscal wrapping [24,25,26,27,28,29]. Biological augmentation techniques are being used to improve outcomes, reduce failure rates, and expand the indications for meniscal repair [20,27,30,31,32,33]. The literature indicates encouraging; however, there are varied outcomes for biologic augmentation in meniscal repair, with the overall efficacy of these techniques remaining inconclusive [29,34,35]. One of the orthobiological techniques is the use of fibrin clot (FC) during meniscal repair procedures [20,25,26,27,28,29,30,34,35]. The FC is an autologous blood-derived product intended to promote healing by releasing a number of bioactive components, including several growth factors. The fibrin molecules of the FC act as a storage and release system for bioactive factors [36,37]. Moreover, the FC can be prepared in a shorter time and at a lower cost than other biologic augmentation procedures, making them a practical choice for meniscus repair augmentation [38]. Unfortunately, there is a lack of high-quality research, such as randomized controlled trials, on the benefits of FC in clinical practice [29]. Furthermore, no study has been conducted to evaluate the efficacy of fibrin clot in the younger patient population, so permitting the exclusion of age-related factors and meniscal degeneration as influencing factors on healing. The aim of this study was to compare clinical and patient-reported outcomes, MRI results, and revision rates (re-operations) following isolated arthroscopic meniscal repair augmented with FC versus without FC in patients under 19 years of age. It was hypothesized that meniscus repair using fibrin clot application would result in lower revision rates, fewer clinical symptoms, higher functional outcomes, and better MRI results at the final follow-up.

## 2. Materials and Methods

The randomized controlled prospective study was started in May 2021 following the approval of the Vilnius Regional Bioethics Committee (Number 2021/51353825), and was registered on the ClinicalTrials.gov platform in December 2023 (ID: NCT06176183) The study was conducted at Vilnius University Hospital Santaros Klinikos. Upon enrollment, all patients’ parents or official caregivers provided informed consent. Patients aged 12 and older were asked to submit additional consent. The inclusion criteria to participate in this study were age under 19 years old, a traumatic isolated full-thickness meniscal tear longer than 1 cm verified by preoperative MRI and arthroscopy, and no previous surgery on the injured knee. Concomitant injuries to the same knee, root or ramp tears, and discoid-type meniscus tears were all excluded due to the need for different surgical procedures and conception of the lesion. Meniscal tears that were only in the white–white (avascular) zone were also excluded from the study due to their poor healing capabilities, which require a separate sample for analysis. Sixty-three patients were recruited and randomly assigned by the computer (Research Randomizer (Version 4.0) [Computer program]. http://www.randomizer.org) to two groups before the meniscus repair procedure. The first group received fibrin clot (FC)-augmented arthroscopic meniscal repair (FC group), while the second group (non-FC group, control group) had the same meniscal repair without additional fibrin clot. Fifty-nine patients (31 males and 28 females) were available for the last follow-up at an average of 12 months after surgery. Twenty-nine (49.2%) patients were included in the FC group, and thirty (50.8%) were in the non-FC group. This study is level II evidence due to its randomized controlled trial design. Demographic data and other characteristics were not significantly different in both groups and are presented in Table 1. The meniscal tears were classified based on location, pattern, vascularity, stability, and complexity. Based on three meniscal anatomic parts—the posterior horn, the body, and the anterior horn—the tears were grouped into those in the posterior horn, the body, and the posterior horn and body regarding injury location.

Meniscal tear types were classified into three groups: longitudinal tears, bucket-handle tears, and complex tears, which consist of several meniscal tear types. As a result, all radial and horizontal tears were combined with other tear types and assigned to the complex tear type group.

Based on the vascularity of the zones where the meniscal tear occurred, two groups were formed: tears that were only in the red–red zone and those that expanded into other zones and reached avascular areas (tears in mixed zones of vascularity). Furthermore, tears were classified according to the stability of the torn fragment in the knee joint as stable or unstable tears. Finally, the tears were also categorized into simple (longitudinal) and demanding (bucket-handle and complex) groups. The distribution of tear types between the groups is shown in Table 1.

### 2.1. Surgical Procedure

All participants in this study underwent arthroscopic meniscal repair surgery. The surgical procedure was identical for both groups, except FC was used in the FC-augmented group.

The surgical procedure was performed by the same dedicated research team—the orthopedic surgeon, who is experienced in arthroscopic surgery, and the anesthesiologist (I. D.), who gave the same regional plus general anesthesia and harvested the blood from FC group participants during the surgical procedure. All patients were operated on lying supine, with a tourniquet around the thigh of the injured limb. The knee was fixed in the knee holder. A traditional two-portal approach was utilized. Three common suturing techniques were employed for meniscus repair, chosen based on the tear type and location, in order to provide optimal fixation and adaptability. Sutures were made either all-inside with internal anchors (Flex fix, Smith & Nephew (Watford, UK) and Fiberstitch, Arthrex, Naples, FL, USA), inside–outside (Meniscus Needles, Arthrex), or outside–inside with a 2–0 fiberwire suture with 18G needles. Based on the usage of repair techniques, the patients were divided into two groups: patients for whom sutures were only made inside the joint anchors were assigned to the all-inside technique group (57.6%), and patients who received a combination of suturing techniques were grouped into the hybrid technique group (42.4%). The relevance of meniscal repair techniques between the groups is demonstrated in Table 1. In the initial phase, the meniscal tear was identified and probed to define its location, size, stability, and overall quality. The tear edges were then renewed with a shaver or rasp, and the tear was anatomically reduced before the sutures were put in. The sutures were positioned in various ways depending on the tear pattern and location, with the primary goal of aligning them vertically to improve fixation strength and restore the meniscus anatomy.

**Figure 1:**
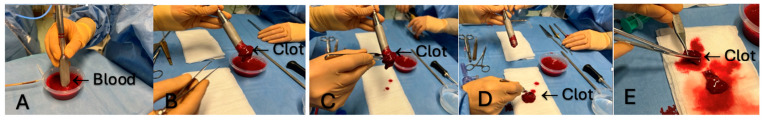
Steps for preparing a fibrin clot. (**A**)—A total of 50 mL of the patient’s blood is stirred for around 15 min. (**B**)—After 15 min, a fibrin clot forms on the instrument’s handle. (**C**)—The formed fibrin clot is gently removed from the instrument’s handle. (**D**)—The fibrin clot is rinsed with water to improve its visualization in the joint. (**E**)—The fibrin clot is cut into smaller pieces, which are then placed into the target area of the joint.

In the FC group, after evaluating the meniscus tear, an anesthesiologist collected 50 mL of blood from the patient’s peripheral vein. Blood was collected into the syringe and placed in a basin. An assistant stirred the blood in the basin with the other instrument’s round iron handle for about 15 minutes, creating a fibrin clot to form on it. After approximately 15 min of stirring, the fibrin clot was then carefully removed from the instrument’s handle and placed on a sterile surgical pad. A total of 5–10 mL of water was usually utilized to rinse the formed clot to enhance clot visualization during arthroscopy. Scissors and picks were employed to cut the fibrin clot into smaller fragments suitable for insertion into the arthroscopic cannula and subsequent transport into the joint. All steps of the preparation of FC are demonstrated in Figure 1. During fibrin clot formation, the meniscal tear edges were renewed, and all or some sutures were applied without being tightened. The one fragment of fibrin clot was then placed in a 5 mm diameter cannula and inserted into the gap between the tear’s margins. The probe was utilized to finalize the location of the fibrin clot, and the suture that could fix that piece of the clot was then tightened together with the meniscus tissue.

**Figure 2:**
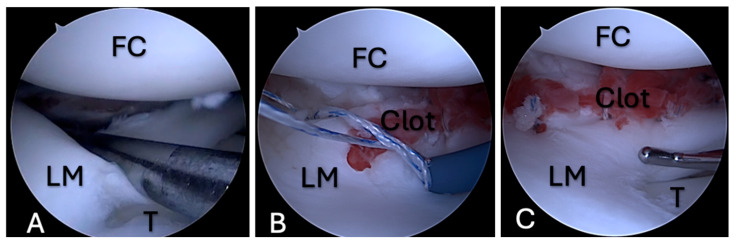
FC application during meniscal repair, case 1. (**A**)—A bucket-handle tear of the lateral meniscus; (**B**)—an FC between the edges of the meniscus tear, with a type of suture that is placed across the tear; (**C**)—an FC between the tear edges that is fixed with sutures. FC—femoral condyle; LM—lateral meniscus; T—tibia.

**Figure 3:**
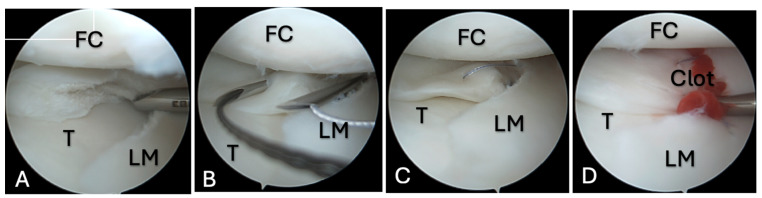
FC application during meniscal repair, case 2. (**A**)—A complex type tear of the lateral meniscus; (**B**)—18G needles used for outside–inside-type sutures; (**C**)—outside–inside-type sutures for meniscus repair; (**D**)—an FC between the tear edges that is fixed with sutures. FC—femoral condyle; LM—lateral meniscus; T—tibia.

**Figure 4:**
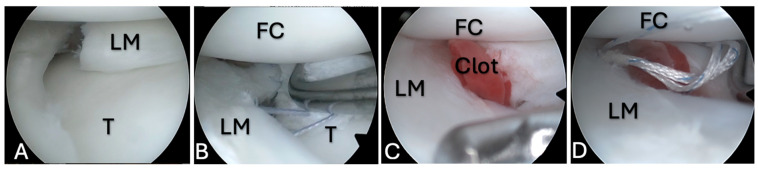
FC application during meniscal repair, case 3. (**A**)—A complex type tear of the lateral meniscus; (**B**)—18G needles used for outside–inside-type suture; (**C**)—outside–inside-type suture for meniscus repair; (**D**)—an FC between the tear edges that is fixed with sutures. FC—femoral condyle; LM—lateral meniscus; T—tibia.

The quantity of fibrin clot pieces required depended upon the extent of the meniscal tear, as was typical with the sutures. Figure 2, Figure 3 and Figure 4 present several cases of meniscus repair augmented with FC.

### 2.2. Postoperative Management and Rehabilitation

The postoperative protocol was the same for both the FC-augmented and non-FC groups. Following the surgery, all patients were allowed partial weight-bearing walking with crutches. The knee was immobilized with a hinged knee brace with 0° of flexion for one week following the surgery, and then the degrees were progressively raised each week until 90°, with an active rehabilitation program beginning six weeks postoperatively. Physical therapy that encourages early quadriceps muscle activation began immediately following surgery. At 1–2 weeks after surgery, the patient began knee flexion exercises from 0° to 90° through a passive range of motion. An active rehabilitation program started six weeks postoperatively. A primary physical medicine and rehabilitation physician (T.A.) supervised rehabilitation programs for all patients. The course of rehabilitation was based on EU-US meniscus rehabilitation consensus [39]. Patients are advised to avoid deep squatting and any squatting exercises for at least four months after surgery. After 4 months, full flexion, squatting, and return-to-normal activities or sports are permitted.

### 2.3. Evaluation and Data Collection

The Paediatric International Knee Documentation Committee (Pedi-IKDC) and Lysholm knee scores were utilized for functional knee assessment, and the Tegner activity scale (TAS) was used to evaluate the patient’s level of sports activity. All scores were completed preoperatively and at the final follow-up, with a median duration of 12 months, in combination with a postoperative MRI examination and clinical evaluation. The Pedi-IKDC score is designed specifically for children and is used to assess knee-related symptoms, function, and sports activity [40]. The Lysholm scale was originally designed to evaluate knee instability symptoms in individuals with knee ligament pathology [41,42]. The TAS is a numerical scale that ranges from 0 (sick leave or disability caused by knee disorders) to 10 (high-level competitive sports) [43].

The clinical evaluation was conducted in person by two researchers at the last follow-up. The clinical assessment involved evaluating pain and swelling and estimating and comparing the range of motion of the knee with the contralateral side. Any reported and objective pain, visible swelling, or difference in range of motion compared to the other extremity were deemed the present symptoms.

### 2.4. MRI Evaluation

All participants in this study underwent postoperative MRI at the last follow-up, with a median of 12 months. The MRI was conducted using 1.5-T MR equipment (SIGNA voyage system). Four main diagnostic sequences were analyzed, including sagittal proton density fast spin echo with fat saturation (Sag-PD FSE FS), sagittal T2-weighted fast spin echo (Sag-T2W FSE), coronal proton density fast spin echo with fat saturation (Cor-PD FSE FS), and coronal T2-weighted fast spin echo (Cor-T2W FSE), with the following parameters. The slices were 3 mm thick, the repetition time ranged from 2863 ms to 4389 ms, the field of view (FOV) was 180 mm, the spacing between slices was 0.3 mm, the number of slices varied from 28 to 31, and the overall scan time was 15 min.

**Figure 5:**
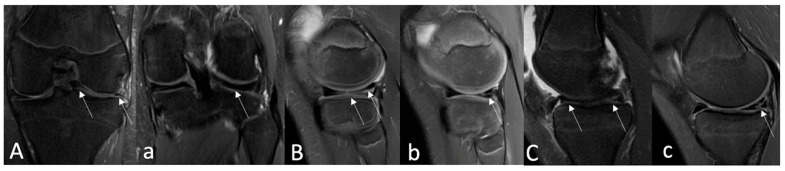
Examples of postoperative MRI assessment based on Stoller and Crues’ three-stage classification. (**A**)—Preoperative coronal PD-FSE, FS MR image shows (arrows) a bucket handle-type lateral meniscus tear in a 16-year-old boy. Twelve-month postoperative coronal (**a**) MR image shows a repaired lateral meniscus and no intrameniscal signal alterations (arrow)—classified as MRI Grade I using Crues and Stroller classification. (**B**)—A preoperative sagittal PD-FSE, FS MR imaging of an 11-year-old boy reveals (arrows) a complex (radial + longitudinal)-type lateral meniscus tear. Sagittal PD-FSE, FS (**b**) MR image obtained 12 months after surgery, where sagittal PD-FSE, FS (**b**) indicates (arrow) a partially healed lateral meniscus with persisting intrameniscal signal changes that do not extend into the joint space—classified as MRI Grade II using Crues and Stroller grading. (**C**)—A 14-year-old girl’s preoperative sagittal PD-FSE, FS MR imaging demonstrates (arrows) a medial meniscus tear of the bucket-handle type. Fourteen-month postoperative sagittal (**c**) PD-FSE FS MR images establish (arrow) an unhealed medial meniscus with signal changes extending within the joint space—classified as MRI Grade III using Crues and Stroller grading. MR—magnetic resonance; FSE—fast spin echo; FS—fat saturation.

Signal alterations on postoperative MRI were rated according to Stoller and Crues’ three-stage classification. Grade 1 was defined as an intrameniscal signal with irregular margins that did not connect or communicate with the articular surface. Grade 2 was described as a linear signal that did not abut or connect with an articular surface. A linear or complex signal intensity that abutted or communicated with an articular surface was classified as Grade 3. In summary, grade 3 was deemed unhealed, grade 2 as partially healed, and grade 1 as fully healed due following MRI assessment [44,45]. A musculoskeletal imaging radiologist (A.B.) and an experienced orthopedic surgeon (V.B.M.) performed the MRI evaluation independently and blinded to functional and clinical evaluation. The intraclass correlation coefficient (ICC) was calculated for interobserver reliability. The overall consensus was reached for each case by both observers. Based on the MRI assessment, only cases with an MRI grade 3 evaluation were considered unsuccessful; therefore, MRI grades 1 and 2 were combined into one group as effectively healed meniscus following MRI, while MRI grade 3 was left separately as unhealed meniscus on MRI images. The postoperative MRI grades for all three groups are shown in Figure 5.

### 2.5. Statistical Analysis

Statistical analysis was performed with SPSS 24.0 IBM. The normality of the variables’ distribution was tested using the Shapiro–Wilk test. Because of the absence of normally distributed variables, nonparametric tests were used. The Mann–Whitney test was employed to compare the groups regarding all functional scores, BMI, the length of follow-up, time to surgery, and age. The chi-square test was utilized to compare the groups based on secondary arthroscopy, clinical evaluation, and MRI findings. The significance level of 0.05 was used to reject the null hypothesis. Measures of central tendency were presented as follows: median (first quartile–third quartile). The ICC degree of agreement was categorized as follows: >0.80, almost excellent reproducibility; 0.61 to 0.80, good reproducibility; 0.41 to 0.60, moderate reproducibility; and 0.40, poor reproducibility. The required minimum sample size was estimated using G*Power analysis software 3.1, which required at least 27 patients per group to detect significant differences with 80% power, a type I error rate of 5%, and a minimum clinically important change (MCID) of 12 points on the Pedi-IKDC scale as the primary outcome measure [46].

## 3. Results

Within the final follow-up, nine (15%) secondary arthroscopies were performed due to an unhealed repaired meniscus, repeated trauma with re-tears, or a clinically significant new meniscus tear. There were two (6.8%) cases in the FC-augmented group and 7 (23.3%) in the non-FC-augmented group, with no statistically significant difference (*p* = 0.145). All secondary arthroscopy cases originate from the demanding (complex and bucket-handle) tear-type subgroup, with the same distribution between the groups based on FC-augmentation (*p* = 0.072).

There were no statistically significant differences in function outcome scores between groups treated with or without FC in meniscus repair. Overall, and in subgroups of meniscus tear types, the FC-augmented group had higher median Pedi-IKDC and Lysholm scale values than the non-FC-augmented group. According to the TAS, 24 (82.75%) out of 29 patients in the FC-augmented groups and 22 (73.3%) out of 30 patients in the non-FC-augmented groups returned to the same level of sport activity at the final follow-up. Table 2 shows the detailed values for comparing the groups’ function scores.

In terms of clinical evaluation, there were no significant differences between the groups except across the unstable and demanding (complex and bucket-handle) tear-type subgroups. In the final follow-up, there were significantly fewer patients with any clinical symptoms in the FC-augmented group among unstable and demanding (complex and bucket-handle) tear-type cases (*p* = 0.012, *p* = 0.041, respectively) (Table 3).

According to the Crues and Stroller classification, the overall postoperative MRI assessment revealed 7 (11.9%) completely healed, 22 (37.3%) partially healed, and 30 (50.8%) unhealed menisci in both groups. Interobserver reliability based on the intraclass correlation coefficient (ICC) was good (ICC = 0.77, 0.65–0.86). The comparison of groups based on MRI results indicated no statistically significant difference between them (Table 4). Notably, the duration of the FC-augmented repair was significantly longer compared to non-FC-augmented meniscus repair (*p* < 0.001, Table 1).

## 4. Discussion

The present clinical trial was designed to compare two groups (experimental and control) and evaluate the efficacy of FC utilization in meniscal repair by analyzing failure rates (secondary arthroscopy), as well as clinical, functional, and radiological (MRI) assessments in young patients. The study’s most significant feature is its design as a prospective clinical trial with a control group registered in ClinicalTrials.gov [47]. To our knowledge, no comparative studies of this type of design have been conducted to assess FC effectiveness during meniscal repair procedures. Furthermore, because our study focused on a specific age group of patients, we dismissed age-related changes in meniscal tissue as degeneration, which also influences healing processes. Since it is known that FC might be particularly useful in less vascularized areas and complex tear types, we conducted the comparison in subgroups. As a result, the most significant finding in our study was that patients who received FC during meniscus repair reported fewer clinical symptoms in the final follow-up across demanding tear types (unstable, bucket-handle, and complex) subgroups. This analysis also revealed that Pedi-IKDC median values in the FC-enhanced group across unstable, complex, and bucket-handle tear-type subgroups are greater, though not significant, with *p* = 0.053 and *p* = 0.066 in both subgroups, respectively, indicating that applying FC may be effective for meniscal healing in particular tear-type of the meniscal tears. We believe that this may be impacted by the small number of participants in each group, and that with an additional number of cases and prolonged follow-up, the groups might demonstrate significant differences. Additionally, this study also reveals the important reoperation number in both groups as two (6.8%) out of twenty-nine in the FC-augmented group and seven (23.3%) out of thirty in the non-FC-augmented group—suggesting superior results were obtained in the FC-enhanced group, though the difference was not significant (*p* = 0.072). All secondary arthroscopies came from demanding tear-type subgroups (bucket-handle and complex) that demonstrated identical distributions across groups based on FC augmentation.

The preservation of meniscus tissue in case of rupture is very important due to the crucial functions of the meniscus in the knee joint [8]. It is especially important in younger individuals for the same reasons, to prevent the knee from early-onset osteoarthritis [9]. Unfortunately, the healing rates vary following the meniscus repairs [16,17,18]. FC has begun to be used to reduce the failure rates [38]. Arzanocky et al. used FC in an animal model to demonstrate the ability of FC to stimulate and support reparative response in meniscus healing [37]. Consequently, several authors have published studies over the past 20 years demonstrating the good results of meniscal repairs using fibrin clots [31,48,49,50,51,52]. Ra et al. showed complete healing on MRI and improved clinical scores in 11 of 12 patients who underwent the arthroscopic inside-out approach using a FC [48]. Van Trommel et al. demonstrated complete healing of a complete radial tear in the posterolateral part of the lateral meniscus by second-look arthroscopy and MRI in five cases [50]. A 90% clinical healing rate was demonstrated in high-risk-of-failure tear types by Davies et al. [31]. Hennings et al. found that the isolated tear failure rate was 41% without the exogenous fibrin clot and 8% with the exogenous clot, a difference that was statistically significant [49]. Nakayama et al. examined a 24-patient case series involving degenerative tear-type repair using FC and concluded that it is highly successful when the knee is well-aligned [51].

Fibrin clots, which are very similar to PRP in terms of content and healing mechanism, serve as a scaffold to fill the defect as well as an initiator and activator of the healing process [38]. They can be made using autologous blood in less time and at a lower cost than PRP, making fibrin clots beneficial in clinical practice and a popular choice for meniscus repair augmentation [38,48]. Nevertheless, there are no standardized protocols for meniscal repair with a fibrin clot, and the successful insertion of a fibrin clot in the target location is challenging due to its adhesion to surgical devices such as probes and arthroscopy instruments [38]. Due to these factors, surgeons are choosing not to apply fibrin clots during meniscal repair [38]. Finally, this additional method is time-consuming, as seen in our study; repairs in the FC-augmented groups required considerably more time in the operating room compared to those without (*p* < 0.001).

Regardless of the study’s good-quality design for the main subject, it contains a few limitations. The patient sample was small, particularly when analyzing subgroups. Although many authors assess meniscus healing rates using further arthroscopy numbers, clinical symptoms, function scores, and MRI imaging, as we also did; nevertheless, these methods are not as accurate as second-look arthroscopy. We did not perform second-look arthroscopy on young patients due to its invasive nature. Furthermore, the use of conventional MRI for meniscus healing is highly subjective for a variety of reasons, as we discussed in our previous research on the subject [53]. MRI arthrography is a more accurate tool, but it is also an invasive procedure [54]. As a result, we acquired 50% of the cases that were considered unhealed on MRI imaging. Additionally, evaluating healing based on clinical symptoms such as pain is highly subjective, as the minimal pain reported during the final follow-up may be attributed to other knee conditions instead of unhealed meniscus tissue. Nonetheless, in this study, the FC-augmented patient group had fewer clinical symptoms at the final follow-up, demonstrating FC’s potential benefit in the management of more demanding tear types and when using this particular way of evaluating the results. Finally, our study has a relatively short follow-up duration; a minimum of two years would be beneficial for more accurate findings.

## 5. Conclusions

Our study is the first randomized clinical trial that investigates how effectively FC works to repair meniscal tears in young patients. The most important finding of this study is that FC augmentation was shown to be beneficial regarding clinical outcomes for patients with unstable, bucket-handle, or complex tears, while for patients with other tear types, it did not demonstrate any advantage. No statistically significant differences were detected among the other outcome measures between the primary groups and subgroups. Differences between FC-augmented and control groups were close to significant in the case of Pedi-IKDC score values, and the secondary arthroscopy rates among unstable, bucket-handle, and complex subgroups encourage the continuation of this study. Therefore, to obtain accurate findings, the study should be enlarged in terms of sample size and follow-up.

## Figures and Tables

**Table 1:** Characteristics of the groups.

Variables	FC-Augmented Group, N = 29 (49.2%)		Non-FC-Augmented Group, N = 30 (50.8%)		*p*-Value
	N (%)	Median (Q1–Q3)	N (%)	Median (Q1–Q2)	
Sex boys/girls	14/15 (48.3/51.7)		17/13 (56.7/43.3)		0.606
Age (years)		16 (14–17)		15 (15–17)	0.779
Meniscal side	15/14		16/14		
medial/lateral	(51.7/48.3)		(53.3/46.7)		0.554
Follow-up (months)		12 (11–14)		12 (11.75–15)	0.383
BMI		21.63 (19.6–23.7)		21.27 (20.2–23.3)	0.862
Location of the tear					
Posterior horn	9 (31.0)		13 (43.3)		0.422
Posterior horn and body	14 (43.8)		13(43.3)		0.796
Body	6 (20.7)		4 (13.3)		0.506
Tear type					
Longitudinal	5 (17.2)		7 (23.3)		0.748
Bucket-handle	9 (31.0)		13 (43.3)		0.422
Complex	15 (1.7)		10 (33.3)		0.192
Time to operation (weeks)		12 (4–24)		12 (4–24)	0.614
Stability of the tear					
stable/unstable	14/15 (48.3/51.7)		13/17 (43.3/56.7)		0.796
Suturing Technique					
all-inside/hybrid	10/19 (34.5/65.5)		15/15 (50.0/50.0)		0.295
Location of the tear					
due to vascularity					
red-red zone	10 (34.5)		9 (30.0)		0.785
mixed zones	19 (65.5)		21 (70.0)		
Operation time (min)		95 (87.5–107.5)		70 (60–90)	<0.001

Abbreviations: Q1—first quartile; Q3—third quartile; BMI—body mass index; N—number of cases.

**Table 2:** The comparison of the groups due to function scores.

Score			FC-Augmented Group, N = 29 (49.2%)	Non-FC-Augmented Group, N = 30 (50.8%)	*p*-Value
	subgroups		Median (Q1–Q3)	Median (Q1–Q3)	
Pedi-IKDC					
Before the repair			53.26 (39.67–61.40)	45.65 (24.32–60.59)	0.585
At the last follow-up			93.47 (85.32–97.27)	86.99 (80.73–96.73)	0.264
Unstable-type tears N = 32			N = 15, 93.47 (89.43–96.73)	N = 17, 85.75 (75.0–95.1)	0.053
Tears in mixed zones of vascularity N = 40			N = 19, 92.39 (76.08–96.73)	N = 21, 85.86 (74.75–95.65)	0.497
Bucket handle-type tears N = 22			N = 9, 92.39 (84.78–97.81)	N = 13, 85.95 (74.45–96.19	0.366
Complex-type tears N = 25			N = 15, 95.65 (91.30–96.73)	N = 10, 86.41 (80.73–96.46)	0.132
Complex- and bucket handle-type tears N = 47			N = 24, 94.01 (89.20–96.73)	N = 23, 85.95 (75–95.65)	0.066
Lysholm					
Before the repair			64 (47.50–74)	65.55 (46.75–73.50)	0.826
At the last follow-up			94 (85–100)	89.50 (84.75–100)	0.607
Unstable-type tears N = 32			N = 15, 94 (85–99)	N = 17, 89 (82.50–97)	0.303
Tears in mixed zones of vascularity N = 40			N = 19, 90 (82–100)	N = 21, 85 (79.50–97)	0.428
Bucket handle-type tears N = 32			N = 9, 94 (88.50–98)	N = 13, 94 (74–99)	0.502
Complex-type tears N = 25			N = 15, 90 (86–100)	N = 10, 89 (84.75–100)	0.593
Complex- and bucket handle-type tears N = 47			N = 24, 94 (86.75–99.75)	N = 23, 89 (84–99)	0.397
Tegner					
Before the repair			4 (3–6)	5.5 (3–7)	0.149
At the last follow-up			4 (2.5–5.5)	4 (2.75–7)	0.379
Unstable-type tears N = 32			N = 15, 4 (3–6)	N = 17, 3 (2–5.5)	0.375
Tears in mixed zones of vascularity N = 40			N = 19, 4 (2–5)	N = 21, 4 (2.5–6.5)	0.391
Bucket-handle-type tears N = 32			N = 9, 4 (3–5)	N = 13, 4 (2–6)	0.845
Complex-type tears N = 25			N = 15, 5 (2–6)	N = 10, 4 (2.75–7)	0.821
Complex- and bucket handle-type tears N = 47			N = 24, 4 (2.75–5.75)	N = 23, 4 (2–6)	0.957

Abbreviations: Pedi-IKDC—Pediatric International Knee Documentation Committee Score; Tegner—Tegner Activity index: N—number of cases; FC—fibrin clot; Q1—first quartile; Q3—third quartile.

**Table 3:** The comparison of the groups according to clinical symptoms.

Clinical Symptom		FC-Augmented Group, N = 29 (49.2%)	Non-FC-Augmented Group, N = 30 (50.8%)	*p*-Value
		N (%)	N/%	
Any pain at the last follow-up				
Overall		8 (27.5%)	11 (36.6%)	0.580
Subgroups				
Unstable-type tears N = 32		1 (6.6%)	6 (35.2%)	0.088
Tears in mixed zones of vascularity N = 40		7 (36.8%)	10 (47.6%)	0.538
Bucket handle-type tear N = 22		1 (11.1%)	5 (38.4%)	0.333
Complex-type tears N = 25		4 (27.6%)	4 (40.0%)	0.667
Complex- and bucket handle-type tears N = 47		5 (20.8%)	9 (39.1%)	0.212
Swelling at the last follow-up				
Overall		4 (13.7%)	3 (10%)	0.706
Subgroups				
Unstable-type tears N = 32		2 (13.3%)	2 (11.7%)	1.0
Tears in mixed zones of vascularity N = 40		3 (15.7%)	2 (9.5%)	0.654
Bucket handle-type tears N = 22		2 (22.2%)	1 (7.6%)	0.544
Complex-type tears N = 25		2 (13.3%)	2 (20%)	1.0
Complex- and bucket handle-type tears N = 47		4 (16.7%)	3 (13%)	1.0
Restriction of ROM at the last follow-up				
Overall		2 (6.8%)	4 (13.3%)	0.671
Subgroups				
Unstable-type tears N = 32		0 (0%)	3 (17.6%)	0.229
Tears in mixed zones of vascularity N = 40		2 (10.5%)	4 (19.0%)	0.664
Bucket handle-type tears N = 22		0 (0%)	2 (15.3%)	0.494
Complex-type tears N = 25		1 (6.7%)	2 (20.0%)	0.543
Complex- and bucket handle-type tears N = 47		1 (4.2%)	4 (17.4%)	0.188
Any clinical symptoms at last follow-up				
Overall		11 (37.9%)	16 (53.3%)	0.299
Subgroups				
Unstable-type tears N = 32		2 (13.3%)	10 (58.8%)	0.012
Tears in mixed zones of vascularity N = 40		9 (47.3%)	14 (66.6%)	0.337
Bucket handle-type tears N = 22		2 (22.2%)	7 (53.8%)	0.203
Complex-type tears N = 25		5 (33.3%)	7 (70.0%)	0.111
Complex and bucket handle-type tears N = 47		7 (29.2%)	14 (60.7%)	0.041

Abbreviations: ROM—range of motion; N—number of cases.

**Table 4:** The comparison of the groups according to postoperative MRI grades.

MRI Grades		FC-Augmented Group, N = 29 (49.2%)	Non-FC-Augmented Group, N = 30 (50.8%)	*p*-Value
		N (%)	N/%	
Group with MRI grade 1 (fully healed) and MRI grade 2 (partially healed) compared to group with MRI grade 3 (unhealed)				
		
Overall		16/13 (55.2/44.8)	13/17 (43.3/56.7)	0.439
Subgroups				
Unstable-type tears N = 32		10/5 (66.7/33.3)	6/11 (35.3/64.7)	0.159
Tears in mixed zones of vascularity N = 40		8/11 (42.1/57.9)	7/14 (33.3/66.7)	0.745
Bucket handle-type tears N = 22		7/2 (77.8/22.2)	6/7 (46.2/53.8)	0.203
Complex-type tears N = 25		6/9 (40.0/60.0)	2/8 (20.0/80.0)	0.402
Complex and bucket handle-type tears N = 47		13/11 (54.2/45.8)	8/15 (34.8/65.2)	0.244

Abbreviations: MRI—magnetic resonance imaging; N—number of cases.

## Data Availability

The data presented in this study are available on request from the corresponding author due to study protocol regulations, corresponding author: viktorija.brogaite@santa.
